# Advanced practice physiotherapy-led triage in Irish orthopaedic and rheumatology services: national data audit

**DOI:** 10.1186/s12891-018-2106-7

**Published:** 2018-06-01

**Authors:** Orna Fennelly, Catherine Blake, Oliver FitzGerald, Roisin Breen, Jennifer Ashton, Aisling Brennan, Aoife Caffrey, François Desmeules, Caitriona Cunningham

**Affiliations:** 1 School of Public Health, Physiotherapy and Sports Science, University College Dublin, Dublin, Ireland; 2 Department of Rheumatology, St. Vincent’s University Hospital, Dublin, Ireland; 3 Health Service Executive, Dublin, Ireland; 4 Department of Physiotherapy, Beaumont Hospital, Dublin, Ireland; 5 Department of Physiotherapy, Adelaide and Meath Hospital, Tallaght Dublin, Ireland; 6 School of Rehabilitation, Faculty of Medicine, University of Montreal, Montreal, Canada; 7 Bone and Joint Clinic, St. Vincent’s University Hospital, Dublin, Ireland

**Keywords:** Physiotherapy, Advanced practice, Triage, Rheumatology, Orthopaedics, Healthcare service research

## Abstract

**Background:**

Many people with musculoskeletal (MSK) disorders wait several months or years for Consultant Doctor appointments, despite often not requiring medical or surgical interventions. To allow earlier patient access to orthopaedic and rheumatology services in Ireland, Advanced Practice Physiotherapists (APPs) were introduced at 16 major acute hospitals. This study performed the first national evaluation of APP triage services.

**Method:**

Throughout 2014, APPs (*n* = 22) entered clinical data on a national database. Analysis of these data using descriptive statistics determined patient wait times, Consultant Doctor involvement in clinical decisions, and patient clinical outcomes. Chi square tests were used to compare patient clinical outcomes across orthopaedic and rheumatology clinics. A pilot study at one site identified re-referral rates to orthopaedic/rheumatology services of patients managed by the APPs.

**Results:**

In one year, 13,981 new patients accessed specialist orthopaedic and rheumatology consultations via the APP. Median wait time for an appointment was 5.6 months. Patients most commonly presented with knee (23%), lower back (22%) and shoulder (15%) disorders. APPs made autonomous clinical decisions regarding patient management at 77% of appointments, and managed patient care pathways without onward referral to Consultant Doctors in more than 80% of cases. Other onward clinical pathways recommended by APPs were: physiotherapy referrals (42%); clinical investigations (29%); injections administered (4%); and surgical listing (2%). Of those managed by the APP, the pilot study identified that only 6.5% of patients were re-referred within one year.

**Conclusion:**

This national evaluation of APP services demonstrated that the majority of patients assessed by an APP did not require onward referral for a Consultant Doctor appointment. Therefore, patients gained earlier access to orthopaedic and rheumatology consultations in secondary care, with most patients conservatively managed.

## Background

A rising prevalence of musculoskeletal (MSK) disorders [1] has impacted on healthcare expenditure and led to increased wait times for orthopaedic and rheumatology services [2, 3]. However, many of these patients with MSK disorders who wait several months or years to see a Consultant Doctor (i.e., Specialist Physician), may not require surgical or medical management. Advanced Practice Physiotherapists (APPs), previously known as Extended Scope Practitioners (ESPs) [4], work in enhanced roles [5, 6] and triage the care of patients waiting for Consultant Doctor appointments, who have usually been deemed non-urgent based on referral information [7]. APPs have been shown to independently manage 55–92% of this selected caseload from orthopaedic waiting lists [8, 9], however, this research has largely been conducted at single sites with a small number of APPs [10]. As APP roles vary between settings, even within the same country [11, 12], multi-site research within each local healthcare context is warranted to ensure these variances are captured [10].

When physiotherapist triage roles were first introduced in the Republic of Ireland, Clinical Specialist Physiotherapists worked only in low back pain clinics [13, 14]. Since 2011, a joint initiative of the National Clinical Programmes for Orthopaedics and for Rheumatology [15] established 24 APP posts in Ireland. The purpose of this new service was to triage the care of a broader MSK population, in 16 of the 33 public adult hospitals with an orthopaedic and/or rheumatology service [16]. The Consultant Doctor or APP screen General Practitioner (GP) referral letters to orthopaedic and rheumatology services, and patients deemed not to require urgent access to Consultant Doctors for surgical or medical interventions, are offered an APP appointment. APPs’ roles include assessment with view to diagnosing, educating, providing advice, and where required, referring onwards to other hospital specialities. Some APPs are also trained in injection therapy but tasks of ordering clinical imaging and listing for surgery are not part of physiotherapy scope of practice in Ireland. However, some hospitals have operating procedures in place to allow APPs arrange imaging and surgery through getting approval and sign-off from a doctor [12].

At the time of APP service introduction in Ireland, in addition to having more than five years of MSK clinical experience and the majority holding postgraduate MSc/PhD degrees, APPs received role-specific training by way of medical team shadowing and mentoring. The APPs were usually co-located with the Consultant Doctors’ outpatient clinics, allowing for medical involvement where required for clinical decisions and administration of injections or surgical listing. If a patient’s condition deteriorated within one year of their initial APP appointment, some hospital sites permitted patients to self-refer (i.e., without an additional GP referral) for an appointment with the APP or Consultant Doctor.

The APP service aims to reduce patient wait times for orthopaedic and rheumatology appointments in a cost-effective manner. However, requirement of onward referrals to Consultants after the APP assessment, or re-referral of patients to orthopaedic/rheumatology services following APP management, could represent additional appointments and thus, costs [17]. Increased throughput of patients due to increased access, may also have knock-on implications for other hospital services such as physiotherapy [18], and monitoring onward referral pathways of patients is therefore critical to facilitate adequate resourcing of services. While a National MSK APP Database captured patient clinical outcomes at the time of new and return/follow-up APP appointments, an additional single-site study was required which specifically identified any patients managed by the APP that later required a re-referral for the same MSK disorder.

This study performed the first evaluation of the MSK APP services utilising the national database. The objectives were to: (i) assess patient wait times from receipt of referral at the hospital to APP appointment; (ii) identify autonomous APP clinical decision-making; (iii) establish clinical outcomes of APP appointments; (iv) and identify re-referral rates of APP service-users at one hospital site.

## Methods

### Ethics

Full ethical approval was received from University College Dublin’s Human Research Ethics Committee (ref. LS-16-04-Fennelly-C), with permission from the National Clinical Programmes and the Ethics and Medical Research Committee at St. Vincent’s University Hospital (SVUH), Dublin.

### Clinical audit

#### National MSK APP database

At the time of establishing the APP service, a National MSK APP Database was devised in collaboration with the APPs, Consultant Doctors, Physiotherapy Managers and the Head of the National Outpatient Department Programme. A 6-month trial of data entry and subsequent reviews by the Data Manager, resulted in minor amendments. Data quality assurance mechanisms included a database training workshop for APPs, monthly review by Physiotherapy Managers at each site, data reports sent to sites for validation by APPs, and data review at quarterly meetings of the national governance team for the MSK initiative. Each APP entered daily data on a local database for all new and follow-up patients attending the orthopaedic and rheumatology APP services. These data were subsequently anonymised and submitted on a monthly basis, in line with data protection policy, to the National Clinical Programmes administration office, and collation by the Data Manager occurred.

In 2014, 22 APPs entered data from 16 hospital sites. At that time, database fields related to clinic (orthopaedic or rheumatology), appointment type (new or return), body region affected by MSK disorder, dates of receipt of GP referral at the hospital and of APP appointment, Consultant Doctor involvement at the APP appointment (via discussion or seeing the patient), clinical investigations ordered, injection administered, surgical listing (surgery or guided injection), physiotherapy referral (Hospital, Community, Private), and other hospital specialty (Orthopaedic Consultant service, Rheumatology Consultant service, Pain clinic, Occupational therapy, Neurosurgery, Neurology, Emergency Department, Geriatrics) referral. If more than one other hospital speciality referral was required, priority was given to recording a Consultant Doctor referral, as this was the focus of the evaluation. A data field for clinical imaging was added to the database in August 2014.

#### Patient re-referral rates

One hospital site [SVUH] was selected for a re-referral rate audit, with a view to potentially extending this across hospital sites, subject to feasibility. At this study site, two APPs worked with six Orthopaedic Consultants and four Consultant Rheumatologists, who screened all GP referral letters. These APPs arranged clinical imaging and surgery via discussion with the Doctor, and one APP was trained in injection therapy. Consecutive patients (*n* = 254) assessed by the APP service during March and April 2014 were identified on the local MSK APP database. An external researcher [OF] extracted the hospital medical numbers of patients managed by the APPs without an onward referral for a Consultant appointment; including those cases where Consultant opinion was obtained at the initial APP appointment. Follow-up of those patient hospital medical numbers on the patient administration system (PAS) identified any further patient contacts with the orthopaedic or rheumatology services (APP or Doctor appointment) within the following two years. Review of patients’ GP discharge letters and/or medical charts determined whether the additional appointment was for the same MSK disorder and body region. As this hospital site permitted patients previously seen by the APP to self-refer for an additional appointment, sources of re-referrals were identified. Consistency in the clinical management decision made at both the ‘re-referral’ and first appointment, were thought to be indicative of appropriate initial management by the APP.

### Data analyses

All data were cleaned, coded and entered into the Statistical Package for the Social Sciences (SPSS), version 20.0. Valid data for new and return patients were analysed utilising descriptive statistics. A subgroup analysis focused on ‘patients referred in 2014’ and evaluated their wait times to reflect current wait times. Patient clinical outcomes were reported across: (1) clinic attended (i.e., orthopaedic or rheumatology); (2) new and follow-up appointments; and (3) body regions of presenting MSK disorder; utilising the cross-tabulation function with categorical variables of clinic, appointment type, and body region. Chi Square [Χ^2^] test for independence was used to compare patient clinical outcomes in orthopaedic versus rheumatology services.

## Results

### National MSK APP database

**Fig. 1:**
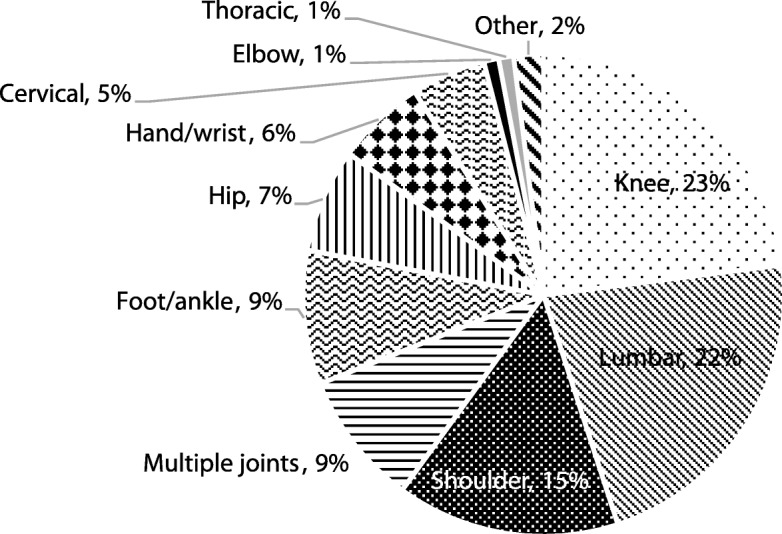
Body regions of the MSK disorders of new patients presenting to the orthopaedic and rheumatology APP services (*n* = 13,367)

Within one year, APPs assessed 13,981 new patients across orthopaedic (84%) and rheumatology (16%) services. Including return (follow-up) patient appointments (*n* = 2596), there were 16,577 patient consultations, with a higher proportion of patients returning for rheumatology as compared to orthopaedics (Table 1). New patients presented most commonly with disorders of the knee, followed by the lumbar-spine, and shoulder (Fig. 1).

**Table 1:** Comparison of clinical outcomes of patients attending advanced practice physiotherapy orthopaedic and rheumatology services

Clinical outcomes	Orthopaedics (*n* = 13,565)	Rheumatology (*n* = 2754)	Chi-Square	*p*-value^ѱ^
	*n* (%)	*n* (%)	Χ^2^	
Return appointments	2064 **(15.2)**	532 **(19.3)**	28.8	< 0.001
Consultant-supported decisions^ф^	2719 **(21.7)**	739 **(28.4)**	55.2	< 0.001
Clinical investigations^Ω^	4023 **(29.7)**	827 **(30.0)**	0.2	0.7
Injection administered	506 **(3.7)**	179 **(6.5)**	43.7	< 0.001
Surgical listing^Χ^	361 **(2.7)**	43 **(1.6)**	11.5	< 0.001
Orthopaedic/Rheumatology Consultant referral^b^	2437 **(19.0)**	343 **(12.9)**	55.6	< 0.001
Physiotherapy referral^a^	5492 **(41.5)**	1022 **(38.0)**	11.4	< 0.001
	**(*n*** **= 5798)**	**(*n*** **= 1211)**		
Clinical imaging^^^	1634 **(28.2)**	231 **(19.1)**	42.5	< 0.001

Unknown clinic (*n* = 258); ^ѱ^ *p* < 0.05 considered significant; ^ф^Consultant-supported decisions valid data for: orthopaedics = 12,541; rheumatology = 2602; ^Ω^ Investigations include imaging; ^Χ^Surgical listing includes guided injections (valid data for orthopaedics =13,564); ^^^Clinical imaging data recorded for latter 5 months; ^b^Consultant referral valid data for: orthopaedics = 12,843, rheumatology =2662; ^a^Physiotherapy includes hospital, primary care, and private services (valid data for: orthopaedics = 13,244, rheumatology = 2692)

#### Wait time

New patients (*n* = 13,456) waited a median time of 167 days (Interquartile Range [IQR] 91–316) for an APP appointment. Median wait time for APP rheumatology services (110 days, IQR 65–217) was less than for APP orthopaedic appointments (177 days, IQR 96–330). APP appointment wait times for patients referred in 2014 (*n* = 6549) was 95 days (IQR 59–139).

#### Independent APP assessment

The APPs made clinical decisions regarding patient management independently at 77.2% (95% CI 76.5–77.9) of all appointments (*n* = 15,189). APPs discussed 16.8% of patient cases with the Consultant, and the Consultant also reviewed a further 6% of patients at the APP appointment. A greater percentage of rheumatology patients required Consultant-supported decisions compared to orthopaedic patients (Table 1). Where Consultant Doctor opinion was obtained at the time of the APP assessment (22.8%; *n* = 3461), 38.9% (*n* = 1346) of those patients were then requested to attend a Consultant appointment.

#### Patient clinical outcomes

##### Clinical investigations

APPs arranged clinical investigations (i.e., imaging, blood tests, neurophysiological tests) for 29.3% of patients. Over the documented five-month period during which imaging had been introduced, image referral occurred for 26.6% of patient cases (Table 2). A greater proportion of patients with multiple joint disorders required clinical investigations, while clinical imaging was ordered most commonly for knee disorders (Table 3). There was no difference in the proportion of patients requiring investigations in orthopaedic versus rheumatology services, but there was for clinical imaging (Table 1).

**Table 2:** Patient clinical outcomes following new and return advanced practice physiotherapy appointments

	Patient appointment type		Total	
Clinical Outcome	New	Return		95% Confidence Interval
	(*n* = 13,981)	(*n* = 2596)	(*n* = 16,577)	
	*n* (%)	*n* (%)	*n* (%)	
Clinical Investigations^a^	4256 **(30.4)**	604 **(23.3)**	4860 **(29.3)**	28.6–30.0
Injections	499 **( 3.6)**	186 **(7.2)**	685 **(4.1)**	3.8–4.4
Surgical listing^b^	263 **( 1.9)**	141 **(5.4)**	404 **(2.4)**	2.2–2.6
Consultant services^c^	2205 **(17.0)**	619 **(23.9)**	2824 **(18.2)**	17.6–18.8
Physiotherapy^d^	6220 **(45.5)**	495 **(19.7)**	6715 **(41.5)**	40.7–42.3
	**(*n*** **= 5976)**	**(*n*** **= 1033)**	**(*n*** **= 7009)**	
Clinical Imaging^e^	1663 **(27.8)**	202 **(19.6)**	1865 **(26.6)**	25.6–27.6

^a^Investigations include imaging; ^b^Surgical listing includes guided injections (valid data for: new = 13,980; total = 15,656); ^c^Consultant services include both orthopaedic and rheumatology Consultants (valid data for new = 12,970; return = 2587; total = 15,557); ^d^ Physiotherapy includes hospital, primary care, and private services (valid data for: new = 13,681; return = 2513; total = 16,194); ^e^Imaging data recorded for latter five months in 2014

**Table 3:** Patient clinical outcomes following advanced practice physiotherapy assessments by body region of presenting musculoskeletal disorder

Clinical Outcome	Body Regions of presenting musculoskeletal disorder									
	Knee	Lumbar-spine	Shoulder	Multiple Joint	Foot/ ankle	Hip	Hand/ wrist	Cervical-spine	Elbow	Thoracic-spine
	(*n* = 3569)	(*n* = 3502)	(*n* = 2429)	(*n* = 1525)	(*n* = 1367)	(*n* = 1139)	(*n* = 1011)	(*n* = 716)	(*n* = 222)	(*n* = 177)
	*n* (%)	*n* (%)	*n* (%)	*n* (%)	*n* (%)	*n* (%)	*n* (%)	*n* (%)	*n* (%)	*n* (%)
Clinical Investigations^a^	1153 **(32.3)**	961 **(27.4)**	578 **(23.8)**	642 **(42.1)**	332 **(24.3)**	418 **(36.7)**	336 **(33.2)**	182 **(25.4)**	60 **(27.0)**	62 **(35.0)**
Injections	129 **(3.6)**	35 **(1.0)**	389 **(16.0)**	21 **(1.4)**	9 **(0.7)**	30 **(2.6)**	44 **(4.4)**	3 **(0.4)**	15 **(6.8)**	1 **(0.6)**
Surgical listing^b^	114 **(3.2)**	18 **(0.5)**	98 **(4.0)**	15 **(1.0)**	19 **(1.4)**	58 **(5.1)**	55 **(5.4)**	2 **(0.3)**	7 **(3.2)**	0 **(0)**
Consultant services^c^	730 **(21.6)**	418 **(12.6)**	364 **(15.6)**	238 **(15.6)**	204 **(15.4)**	345 **(32.9)**	257 **(25.9)**	71 **(10.2)**	42 **(19.3)**	17 **(10.6)**
Physiotherapy^d^	1565 **(44.7)**	1393 **(40.9)**	988 **(41.8)**	646 **(42.5)**	630 **(46.9)**	393 **(36.7)**	165 **(16.8)**	369 **(52.9)**	88 **(40.9)**	86 **(50.0)**
	**(*n*** **= 1573)**	**(*n*** **= 1461)**	**(*n*** **= 1021)**	**(*n*** **= 749)**	**(*n*** **= 586)**	**(*n*** **= 565)**	**(*n*** **= 392)**	**(*n*** **= 296)**	**(*n*** **= 77)**	**(*n*** **= 81)**
Clinical Imaging^e^	491 **(31.2)**	387 **(26.5)**	221 **(21.6)**	247 **(33.0)**	139 **(23.7)**	191 **(33.8)**	59 **(15.1)**	75 **(25.3)**	16 **(20.8)**	21 **(25.9)**

^a^Investigations include imaging; ^b^Surgical listing includes guided injections (valid data for: knee = 3568); ^c^Consultant services include both orthopaedic and rheumatology (valid data for knee = 3379; lumbar spine = 3313; shoulder = 2334; multiple joint = 1522; foot/ankle = 1326; hip = 1050; hand/wrist = 992; cervical-spine = 698; elbow = 218; thoracic-spine = 160); ^d^Physiotherapy includes hospital, primary care, and private services (valid data for: knee = 3500; lumbar spine =3407; shoulder = 2361; multiple joint = 1520; foot/ankle = 1342; hip = 1070; hand/wrist = 981; cervical-spine = 698; elbow = 215; thoracic-spine = 172); ^e^Imaging data recorded for latter five months in 2014

##### Intra-articular injections

Only 4.1% of patients received an injection at their APP appointment from either the APP or Doctor (Table 2), and more than half of these were for shoulder disorders (Table 3). Injections were administered to a greater proportion of rheumatology than orthopaedic patients (Table 1).

##### Surgical intervention

At the APP appointment, 2.4% of patients were listed for surgery (including guided injections [0.3%]) by either the APP or Consultant Doctor (Table 2), most commonly patients with hand/wrist disorders (Table 3). A larger proportion of surgical listing occurred in orthopaedics than rheumatology (Table 1).

##### Onward referrals

While previously Doctors assessed all patients, now only 18.1% of patients required an onward referral to Orthopaedic or Rheumatology Consultant services (Table 2), most commonly patients with hip disorders (Table 3). A significantly smaller proportion of rheumatology patients required a Consultant referral, compared to orthopaedic patients (Table 1).

In addition to onward Consultant referrals, a further 3.6% of patients (Table 2) received an onward referral to other hospital specialities (Table 4). Physiotherapy, the most common onward referral (41.5%), included referral to hospital, community, and private physiotherapy (Table 4). A significantly greater proportion of orthopaedic than rheumatology patients were referred to physiotherapy (Table 1). Of note, APP referral of patients with hand/wrist disorders for physiotherapy was low compared to other body regions (Table 3).

**Table 4:** Onward referral destinations of patients following advanced practice physiotherapy assessments

Referral destination	Patients
	*n* (%)
Primary care physiotherapy^a^	3568 **(22.0)**
Hospital physiotherapy^a^	3130 **(19.3)**
Orthopaedic Consultant services^b^	2453 **(15.8)**
Rheumatology Consultant services^b^	365 **(2.3)**
Pain clinic^b^	140 **(0.9)**
Occupational therapy^b^	97 **(0.6)**
Neurosurgery^b^	23 **(0.1)**
Private physiotherapy^a^	17 **(0.1)**
Neurology^b^	8 **(< 0.1)**
Emergency department^b^	3 **(< 0.1)**
Geriatrics^b^	1 **(< 0.1)**
Other^b^	291 **(1.9)**

Database permitted recording of physiotherapy in one column and all other hospital specialities in another. Priority was given to recording of a Consultant Doctor referral. ^a^Physiotherapy valid data = 16,194; ^b^Other Hospital Specialty valid data = 15,557

### Re-referral rate audit

Over two months at a single hospital site (SVUH), APPs assessed and managed the care of 184 patients without onward referral to Consultant Doctors. Twenty (10.9%; 95% CI 6.4–15.4) of those patients were re-referred to orthopaedic or rheumatology services for the same MSK disorder, within two years of their initial appointment, and 12 (6.5%; 95% CI 2.9–10) within one year. Three of those patients (15%) had had Consultant involvement at the time of their initial appointment and half of those re-referrals were for knee disorders (*n* = 10). Seven patients self-referred for the additional appointment as opposed to a doctor referral (*n* = 10); with the re-referral pathway of three patients unclear. Seventy percent (*n* = 14) of re-referred patients had no change made to their clinical care pathway, including four patients seen by a Consultant Doctor at their return appointment. Changes to clinical management included surgical listing, following a Consultant review (*n* = 2), and APPs arranged MRIs for two patients, an injection for one patient and a neurological referral for another patient.

## Discussion

This is the first national evaluation of MSK APP services and it demonstrated that this new model of service delivery facilitated APP independent assessment and clinical decision making regarding the care of patients from Consultant Doctor orthopaedic and rheumatology waiting lists. Nearly 14,000 patients accessed specialist orthopaedic and rheumatology reviews via the APP service within one year. Therefore, these patients gained more timely access to orthopaedic and rheumatology services, compared to national wait time figures of over 12 months for some Consultant services [16]. While APP services have existed for longer in orthopaedic and spinal triage clinics [8, 9, 11, 13], this multi-site study demonstrated that APPs managed over 80% of patients with a variety of MSK disorders across the two specialities of orthopaedics and rheumatology, without onward referral to a Consultant. This allowed Consultant Doctors to prioritise their time for more complex or surgical patients [9].

Referral for physiotherapy treatment was the most common clinical outcome from the triage process, a similar finding of previous research in orthopaedic settings [8, 19]. Despite this, Blackburn et al. [20] noted that the majority of patients attending their APP orthopaedic service, had not had prior physiotherapy treatment, which potentially could have precluded some patients being placed on secondary care waiting lists. However, further resourcing of physiotherapy would be required to support larger throughput of patients in primary care.

Increased autonomy of APPs to order diagnostic imaging and administer injections may potentially reduce burden on Consultant Doctors’ time. Changes to legislation in Ireland to permit physiotherapists to order imaging, as well as further provision of training on imaging interpretation and injection administration, would allow APPs to work more autonomously [12]. Concerns that placing APPs in such roles might drive higher usage of diagnostic imaging appeared unfounded, with APPs recommending imaging for less than 30% of patients in the current study, and previous research in orthopaedic settings demonstrating that APPs arranged similar proportions of imaging as doctors [21, 22].

While the Irish Health Service Executive (HSE) aims to manage non-surgical patients in the primary care setting, many of these patients still receive secondary care specialist referrals [23]. The results of this study may support the relocation of APP services to primary care, as in the UK and Sweden [24, 25]. However, close proximity of the Consultant and clinical investigations in hospitals may alleviate any potential barriers to Consultant referrals [26], and concerns of delayed patient management [27]. Co-location of APP and Consultant clinics may also reduce referrals to Consultant services, as discussion on clinical management can occur on the day of the appointment. For example, rheumatology patient management required more Consultant-supported decisions, but these data showed empirically that only a small proportion were then referred to Consultant services.

While well documented in orthopaedics settings [7], encouragingly review of the re-referral rate in the APP rheumatology service at one site, did not identify any medically-urgent re-referrals. Additionally, allowing patients to self-refer for another MSK appointment ensures rapid review if required, while increasing patient satisfaction [28], and this did not over-burden the service as few patients utilised this access route. Australian and UK studies noted similar re-referral rates [8, 17], which could be attributed to deterioration in conditions. Re-referral evaluations should now be extended to include other sites to capture geographic or case-mix discrepancies, and perhaps also identify any re-referrals to other hospitals and changes in the patients’ condition.

Limitations are known to exist with such large administrative databases including missing or invalid data fields [29], and for this study, complete data for each field were available for 85% of patient cases. Ongoing monitoring of the National MSK Database is recommended as the APP service becomes more embedded in the Irish orthopaedic and rheumatology services, and future data collection should include information on prior MSK service interactions, linking of individual patients’ new and return appointments, and allow selection of multiple onward specialty referrals. Additionally, a longitudinal follow-up of the patient outcomes is recommended to determine the appropriateness of APP management through capturing treatment effectiveness, validity of diagnosis, or subsequent surgical conversion rates.

## Conclusion

The APP service allowed 13,981 new patients to access orthopaedic and rheumatology consultations within one year, and the majority of patients were independently assessed by the APPs. This first national evaluation of patient clinical care pathways from APP services identified that less than 20% of patients required a Consultant Doctor referral following an APP assessment. This improved patient access to orthopaedic and rheumatology services and thus, clinical management options. Overall, these findings support the APP model of care for patients in orthopaedic and rheumatology settings.

## Abbreviations

95% CI: 95% Confidence Interval
APP: Advanced Practice Physiotherapist
IQR: Interquartile range
MSK: Musculoskeletal
Χ^2^: Chi-square test
